# Relationship between frailty, according to three frail scores, and clinical and laboratory parameters of the geriatric patients with type 2 Diabetes Mellitus

**DOI:** 10.1590/1806-9282.20220271

**Published:** 2022-08-19

**Authors:** Seyma Akan, Gulali Aktas

**Affiliations:** 1 Abant Izzet Baysal University Hospital, Department of Internal Medicine – Bolu, Turkey.

**Keywords:** Morbidity, Mortality, Hospitalization

## Abstract

**OBJECTIVE:**

Type 2 diabetes mellitus is associated with significant morbidity and mortality. The term “frailty in the elderly” has become increasingly important with the increase of the elderly population, especially in diabetic subjects. Frailty is established by various scoring scales, such as Edmonton, Frail, and Prisma-7 scores. We aimed to evaluate the association between frailty and clinical and laboratory parameters of the geriatric type 2 diabetic patients.

**METHODS:**

Diabetic patients over 65 years of age who presented to outpatient internal medicine clinics of our institution between June 2020 and January 2021 were enrolled to the study. Edmonton, Frail, and Prisma-7 scores were administered to the subjects. Study parameters were compared between well-controlled and poorly controlled diabetic groups according to diabetes control level and between frail and non-frail groups, according to each frailty scores.

**RESULTS:**

Frailty according to Edmonton score was associated with increased risks of hospitalization (p=0.005) and mortality (p=0.02). Frailty according to frail score was associated with increased risk of hospitalization (p=0.009). Frailty according to Prisma-7 score was associated with increased risk of mortality (p<0.001).

**CONCLUSION:**

We suggest that Edmonton frail score is superior to Frail and Prisma-7 scores in determining frailty in geriatric patients with type 2 diabetes mellitus, since it is associated with both increased risk of hospitalization and mortality within 6 months.

## INTRODUCTION

Type 2 diabetes mellitus (T2DM) and related disorders are among the top 10 of the all-cause mortality worldwide. Frailty is a novel term that refers to decline in physical capacity and cognitive functions in the elderly, which could be accelerated by T2DM. Falls, dementia, delirium, increased hospitalization, and increased mortality are associated with frailty^1^. Even minor stress can cause serious morbidity and mortality in frail individuals^1^. Thus, many tools have been developed to assess frailty reliably, including Edmonton frail scale^2^, Prisma-7 score^3^, and Frail scoring system^4^.

In present study, we aimed to assess possible association between frailty (according to each of Edmonton frail scale, Prisma-7 score, or Frail score) and clinical and laboratory parameters in geriatric subjects with T2DM.

## METHODS

### Design, setting, and population

Patients diagnosed with type 2 diabetes who presented to Bolu Abant İzzet Baysal University Hospital between June 2020 and January 2021 were included in the study. Subjects under 65 years of age or with active infection or inflammatory disease, who had trauma or surgery in the last 1 month, patients with malignancy, and those who did not want to participate were excluded from the study. By questioning the history of each patient (e.g., diabetes duration, medications used, and concomitant diseases), physical examination findings, blood pressure, and height-weight measurements were recorded. Body mass index (BMI), HbA1c, complete blood count, urea, creatinine, glomerular filtration rate (GFR), serum electrolytes, albumin, aspartate (AST) and alanine (ALT) aminotransferases, lipoprotein fractions, and spot urine albumin/creatinine values were recorded. The patients were grouped as well or poorly regulated diabetics according to their HbA1c levels (i.e., HbA1c≤7.5 well-regulated; HbA1c>7.5 poorly regulated).

Edmonton, Frail, and Prisma-7 frailty scales were applied to the patients face-to-face using a questionnaire. Patients were grouped according to whether or not they were frail for each scale (14–18). Those who scored 0–7 on the Edmonton vulnerability scale were not frail and those who scored 8–17 were considered frail. Those who scored 0–2 on the frail scale were considered not frail, while those who scored 3–5 were considered frail. Those who scored less than 3 on the Prisma-7 vulnerability scale were considered not frail, while those who scored 3 and more than 3 were considered frail. Laboratory parameters and anthropometric measurements were recorded. The patients or their relatives were contacted again 6 months after participating in the study, and the mortality and morbidity status requiring hospitalization during this period were recorded. General characteristics, laboratory values, other parameters, and frailty scores of the patients were compared between those well and poorly controlled T2DM groups as well as between the frail and non-frail patients according to each of Edmonton, Prisma-7, and Frail scores.

### Statistical analyses

Study data were analyzed using the SPSS statistical software (SPSS 15.0, IBM Co., Chicago, IL, USA). The Kolmogorov-Smirnov test was used to determine whether the data fit into the normal distribution between the study groups. Normally distributed data were analyzed by t-test and expressed as mean±standard deviation (SD). Data that did not fit the normal distribution were compared with the Mann-Whitney U test and expressed as the median (min–max). Intergroup comparison of categorical variability was performed with the chi-square test and expressed as n (%). The sensitivity and specificity of the study variables in predicting mortality or morbidity were evaluated by ROC analysis test. A p<0.05 value was accepted for statistical significance level.

## RESULTS

A total of 100 diabetic subjects were enrolled to the study, of which 34 (34%) were women and 66 (66%) were men. Frailty scores, number of hospital admissions within 6 months, and mortality rates of the well and poorly controlled DM groups were not statistically different (p=0.754 and p=1, respectively). Notably, 28% of the study population was frail according to Edmonton scale, 44% according to Frail score, and 19% according to Prisma-7 score. There were no gender difference between frail and non-frail groups (p=0.17 for Prisma-7; p=0.09 for Frail; and p=0.49 for Edmonton scores). The association between frailty according to the Edmonton, Frail, and Prisma-7 scales and mortality and the number of mortality is shown in Table 1. The laboratory parameters of the frail and non-frail subjects according to the Edmonton scale, Frail score, or Prisma-7 score were summarized in Table 2.

**Table 1.** Relationship of frailty according to Edmonton, Frail, and Prisma-7 scales with 6-month mortality and number of hospitalizations.

	Mortality (%)	p	Number of hospitalizations**	p
Frail according to Edmonton scale (n=28) Non-frail according to Edmonton scale (n=72)	n=5 (18) n=3 (4)	0.02	1 (0–4) 0 (0–4)	0.005
Frail according to Frail score (n=44) Non-frail according to Frail score (n=56)	n=6 (14) n=2 (4)	0.07	1 (0–4) 0 (0–4)	0.009
Frail according to Prisma-7 score (n=19) Non-frail according to Prisma-7 score (n=81)	n=6 (32) n=2 (3)	<0.001	1 (0–3) 0 (0–4)	0.06

**Number of hospitalizations were expressed as median (min–max) (n=number of subjects). Significant p-values were expressed as bold characters.

**Table 2.** Laboratory parameters of the frail and non-frail subjects according to Edmonton, Frail, and Prisma-7 scores.

	Frail according to Edmonton scale** Non-frail according to Edmonton scale**	p	Frail according to Prisma-7 score** Non-frail according to Prisma-7 score**	p	Frail according to Frail score Non-frail according to Frail score	p
Albumin (g/L)	3.9 (2.9–4.5) 4 (2.7–5)	**0.01**	3.7 (2.9–4.5) 4 (2.7–5)	**0.003**	3.9 (2.7–4.6) 4 (2.9–5)	**0.03**
Urea (mg/dL)	55 (28–128) 41 (17–146)	**0.02**	56 (28–126) 41 (17–146)	**0.01**	52 (28–146) 41 (17–118)	**0.02**
Creatinine (mg/dL)	1.2 (0.75–2.4) 1 (0.65–5.5)	**0.048**	1.2 (0.76–2.2) 1 (0.65–5.5)	**0.08**	1.1 (0.7–5.5) 1 (0.7–3)	**0.24**
GFR (mL/dL/1.73 m^2^)	55 (21–94) 67 (10–99)	**0.03**	54 (21–94) 66 (10–99)	**0.04**	56±23 66±21	**0.03**
Hb (g/dL)	12.2±2.2 12.8±1.8	**0.21**	11.7±2.3 12.8±1.8	**0.02**	12±2 13.1±1.7	**0.004**
LDL (mg/dL)	83±29 101±44	**0.04**	82 (28–174) 90 (28–255)	**0.13**	91±42 100±41	**0.31**
Triglyceride (mg/dL)	133 (66–322) 146 (59–512)	**0.13**	115 (66–322) 155 (59–512)	**0.008**	127 (66–512) 166 (59–352)	**0.01**
Total cholesterol (mg/dL)	153 (67–236) 171 (65–326)	**0.03**	148 (67–236) 165 (65–326)	**0.03**	164±50 179±51	**0.14**

**Data with normal distribution were expressed as mean±SD and data that did not fit the normal distribution were expressed as the median (min–max). Significant p-values were expressed as bold characters.

Frail subjects according to either Edmonton scale (p=0.002), Frail score (p=0.004), or Prisma-7 score (p=0.004) were older than those who were not frail. Waist circumference of the frail group according to Edmonton scale was increased compared to the non-frail patients (p=0.04). BMI of the frail population according to Frail score was increased compared to the non-frail patients (p=0.04).

**Figure 1.**
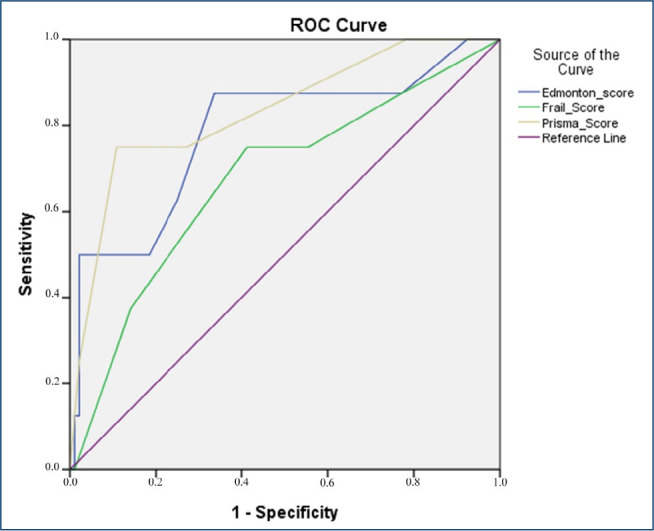
Receiver operative characteristics (ROC) curves of Edmonton, Frail, and Prisma-7 scales in predicting mortality.

The sensitivity and specificity of the Edmonton, Frail, and Prisma-7 scales in predicting mortality were evaluated by ROC analysis. The Edmonton frailty scale (7 points and above) showed mortality with 88% sensitivity and 66% specificity (AUC=0.78, p=0.008, 95%CI 0.6–1.0). Frail scale (3 points and above) predicted mortality with 75% sensitivity and 59% specificity (AUC 0.67; p=0.1; 95%CI 0.5–0.9). Prisma-7 score (4 points and above) showed mortality with 75% sensitivity and 89% specificity (AUC 0.83; p=0.002; 95%CI 0.7–1.0) (Figure 1).

## DISCUSSION

Main findings of present study were

1. diabetic regulation, either poor or well control, was not associated with hospitalization, mortality nor frailty in elderly,
2. frail subjects according to Edmonton score had increased mortality and hospitalization compared to non-frail subjects while frail subjects according to Frail scale showed association only with hospitalization and those frail subjects according to Prisma-7 score showed association only with mortality, and
3. Edmonton score greater than 6 points had the best sensitivity and Prisma-7 score over 3 points had the best specificity in predicting mortality.

We found that diabetic control level was not associated with frailty in diabetic subjects over 65 years. This was valid for determination of frailty with all three frailty scales. Being diabetic, well or poorly controlled, alone will not influence frailty. In the literature, there are studies revealing that poorly controlled diabetes leads to frailty by causing loss of functionality in many organs and systems with its accompanying complications^5^. HbA1c levels of the diabetic subjects were associated with frailty in Bilgin et al.’s study^6^. However, low HbA1c levels are also associated with increased mortality and risk of hospitalization in elderly patients with T2DM. In addition, Yanagita et al. reported that tight glycemic control was a risk factor for frailty in elderly patients^7^.

Studies have shown that every 1 g/dL decrease in hemoglobin concentration increases the risk of frailty approximately 2 times according to the Frailty in Brazilian seniors (FIBRA) study conducted in Brazil^8^. Lower hemoglobin values in older participants were reported in our study, which is in line with the literature data. In addition, we found that participants with high frailty scores, according to all three scales, have more serious anemia.

Roshanravan et al reported the prevalence of frailty was 14% in subjects with stages 1–4 chronic kidney disease, which was almost twice of the prevalence of frailty in the control group without kidney disease^9^. Glomerular filtration rate (GFR) levels were lower and urea values were higher in frail subjects according to Edmonton scale, Frail score, or Prisma-7 score compared to non-frail subjects.

A decrease in lean body mass causes sarcopenia and frailty. Studies have shown that there was an inverse relationship between serum albumin level and frailty. Low serum albumin level was suggested as an independent risk factor for frailty^10^. Similarly, we observed lower serum albumin levels in frail subjects according to all of three frailty scales compared to non-frail diabetics in present study.

Defining frailty and taking appropriate measures to prevent it become more important recently than before as the average life expectancy is getting longer for all populations^1,11^. Detection of the frail elderly enables to predict prolonged hospitalization and mortality, even when faced with moderate to mild stress situations^12^. In our study, unlike the Prisma-7 and Frail scales, frailty according to Edmonton score was found to be associated with both increased mortality and risk of hospitalization. Similarly, in a study from Vietnam, frailty according to Edmonton score was found to be associated with both prolonged hospitalization and 6-month mortality^13^. Frailty according to Frail score was reported to be associated with increased risk of hospitalization but mortality in present study. In accordance, Chong et al. found that frailty according to the Frail score predicted mortality in hospitalized patients successfully^14^.

It is a fact that malnutrition and reduced muscle mass cause frailty. However, studies have also shown that excess weight also cause deterioration in metabolic balance and inactivity, paving the way for frailty^15^. Villareal et al. reported that physical exercise and weight loss can reduce frailty in older obese individuals^16^. A total of 4984 subjects older than 60 years were studied and higher body fat ratio and waist circumference measurements were found in frail subjects compared to non-frail age and sex-matched controls^17^. Moreover, Hubbard et al. suggested that increased waist circumference and abdominal fat were associated with frailty, even in low-weight individuals^18^. Consistently, we reported that abdominal obesity was more common in frail group compared to the non-frail diabetics in present study.

Increased 6- and 12-month mortality has been reported in frail subjects according to Prisma-7 score compared to non-frail elderly^3^. Similarly, in the present study, mortality was more common in frail group according to Prisma-7 score than the mortality in non-frail group.

We found lower AST and ALT values in patients who were frail according to Edmonton scale compared to non-frail group. The reduction in transaminase levels is thought to be associated with frailty as a result of malnutrition. Considering that pyridoxine (vitamin B6) is a cofactor for transaminases, a decrease in AST and ALT levels is expected in B6 deficiency^19^. Le Couteur et al. revealed that ALT may be a new biomarker of aging and is associated with frailty^20^.

Frailty is associated not only with T2DM, as presented in our work, but also with other chronic conditions, such as cancer^21,22^.

The fact that our study was a single-center study and carried out in a relatively small cohort limits the generalization of its results. However, to the best of our knowledge, it is the first study in which three separate frailty scales were evaluated in geriatric diabetic subjects and that observed the association of frailty scores with hospitalization and mortality.

## CONCLUSION

We suggest that Edmonton frail score is superior to Frail and Prisma-7 scores in determining frailty in geriatric patients with T2DM, since it is associated with both increased risk of hospitalization and mortality within 6 months.
